# Responses of Nucleus Tractus Solitarius (NTS) early and late neurons to blood pressure changes in anesthetized F344 rats

**DOI:** 10.1371/journal.pone.0169529

**Published:** 2017-04-06

**Authors:** Jenya Kolpakova, Liang Li, Jeffrey T. Hatcher, He Gu, Xueguo Zhang, Jin Chen, Zixi Jack Cheng

**Affiliations:** 1 Burnett School of Biomedical Sciences, College of Medicine, University of Central Florida, Orlando FL, United States of America; 2 Department of Neurology, Henry Ford Hospital, Detroit, Michigan, United States of America; Harbin Medical University, CHINA

## Abstract

Previously, many different types of NTS barosensitive neurons were identified. However, the time course of NTS barosensitive neuronal activity (NA) in response to arterial pressure (AP) changes, and the relationship of NA-AP changes, have not yet been fully quantified. In this study, we made extracellular recordings of single NTS neurons firing in response to AP elevation induced by occlusion of the descending aorta in anesthetized rats. Our findings were that: 1) Thirty-five neurons (from 46 neurons) increased firing, whereas others neurons either decreased firing upon AP elevation, or were biphasic: first decreased firing upon AP elevation and then increased firing during AP decrease. 2) Fourteen neurons with excitatory responses were activated and rapidly increased their firing during the early phase of AP increase (early neurons); whereas 21 neurons did not increase firing until the mean arterial pressure changes (ΔMAP) reached near/after the peak (late neurons). 3) The early neurons had a significantly higher firing rate than late neurons during AP elevation at a similar rate. 4) Early neuron NA-ΔMAP relationship could be well fitted and characterized by the sigmoid logistic function with the maximal gain of 29.3. 5) The increase of early NA correlated linearly with the initial heart rate (HR) reduction. 6) The late neurons did not contribute to the initial HR reduction. However, the late NA could be well correlated with HR reduction during the late phase. Altogether, our study demonstrated that the NTS excitatory neurons could be grouped into early and late neurons based on their firing patterns. The early neurons could be characterized by the sigmoid logistic function, and different neurons may differently contribute to HR regulation. Importantly, the grouping and quantitative methods used in this study may provide a useful tool for future assessment of functional changes of early and late neurons in disease models.

## Introduction

The nucleus tractus solitarius (NTS) barosensitive neurons receive direct synaptic inputs from primary baroreceptor afferent fibers that convey arterial pressure (AP) information for cardiovascular regulation [1]. Baroreceptor afferent inputs are integrated in the NTS, which contains secondary, higher order and output neurons [2]. The output neurons relay the processed input to other central sites involved in regulation of cardiovascular function. NTS output neurons send axons to the nucleus ambiguus as well as caudal and rostral ventrolateral medulla to regulate cardiac function and AP [1, 3–7].

Previously, numerous different types of NTS barosensitive neurons have been identified. Seagard et al. found two functional types of NTS neurons in mongrel dogs responding to pressure ramp increase: sudden-onset rapid-adapting neurons and slow onset non-adapting neurons [8]. Paton et al. identified the adaptive, non-adaptive neurons, and prolonged excitation NTS neurons responding to AP changes in rats [9]. Rogers et al. reported that NTS neurons not only encode mean arterial blood pressure (MAP), but are sensitive to the rate of AP change [10]. It was later confirmed by Zhang and Mifflin that different rates of AP change may result in different neuronal responses in the same NTS neurons [11]: a fast AP increase rate may lead to a higher response compared to a slow rate increase even though the peak of AP elevation is the similar. Deuchars et al. found that NTS barosensitive neurons are both physiologically and morphologically heterogeneous [12]. Interestingly, NTS subthreshold neurons which can only be activated by aortic nerve stimulation after excitatory amino acid application [13]. These neurons are in the “reserved state” and can be activated under certain conditions [13–14] Thus far, a variety of heterogeneous NTS barosensitive neurons have been identified and their properties have been described.

NTS barosensitive neurons vary considerably in terms of their basal activities and their peak discharge response to AP changes relative to basal activity (ranging from 5 to 80 Hz, see Fig 2 in [11]), the firing patterns, and time course of the response. Under different experimental conditions, NTS barosensitive neurons may vary in their responses. For example, the peak discharge of NTS neurons in response to AP changes depends on the rising rate of the AP elevation [10–11]. Therefore, in order to study the functional changes of different populations of barosensitive NTS neurons in disease models, it is critically important to first define the subpopulations of NTS neurons according to their firing properties, and then to quantify their firing activity-AP relationship, and finally to examine the time-course of their activity upon AP changes at the same rate. In this study, we made extracellular recordings of the changes in activity of single NTS neurons in response to AP changes that were increased to the AP plateaus at the similar rate and maintained at these plateaus for 5–7 seconds by partial occlusion of the descending aorta in anesthetized rats. The goal was to first classify the neurons which have similar firing properties, and then establish a method by which the discharge in response to AP changes can be quantified. In addition to the discharge peak, we also measured the time course of discharges relative to AP changes. Since the sigmodal logistic function curve has previously been effectively used in characterizing baroreceptor afferent nerve activity [15–16], we also attempted to use the sigmoid logistic function curve and quantify NTS neurons’ discharges in response to AP changes. NTS neurons with different characteristic firing properties in response to AP changes were identified: excitatory (majority), inhibitory or biphasic (minority). In the excitatory group, the neurons were further divided into early and late neuron subgroups according to their response time course. The early neurons rapidly increased their firing during the early phase of MAP rising, whereas late neurons did not increase their firing until the ΔMAP reached near/after the peak. We found that the early neurons have a significantly higher firing rate in response to AP elevation than the late neurons. The early NA-MAP relationship, but not the late NA, could be quantitatively characterized by six parameters in the logistic function curve. Finally, we correlated early and late neurons firing activity with heart rate (HR) reduction and suggested that early and late neurons may differently contribute to HR regulation.

## Materials and methods

### Animals

Fischer 344 (F344; 3–4 month) male rats from Harlan were used. 2–3 Rats were housed in a cage upon arrival at University of Central Florida animal facility. The room light and dark cycles were set at 12:12 h (6:00 AM to 6:00 PM). Temperature was kept at 22–24°C. Water and chow were freely accessible (24 hours/day/ 7 day per week). At the end of the experiments, rats were euthanized by an injection of a lethal dose of pentobarbital (100mg/kg). Tissues including the heart and brain and were removed after the animals’ heart beats had completely stopped. Procedures were approved by the University of Central Florida Animal Care and Use Committee and followed the guidelines established by the National Institutes of Health.

### Surgical procedure

The surgical procedure was similar to what has been previously described [16–20]. Briefly, rats were initially anesthetized with sodium pentobarbital (50 mg/kg i.p.). Body temperature was monitored and controlled by a rectal probe and maintained at 37 ± 1°C with a homeostatic blanket (Harvard, Holliston, MA). Supplemental doses (5 mg/kg i.v.) of sodium pentobarbital were administered as needed to prevent eye blink and withdrawal reflexes to toe-pinch as needed. When the animals were no longer responsive to toe-pinch, we performed the following surgical procedures. Animals were given a tracheal intubation and oxygen-enriched room air was provided through ventilation. The femoral vein was cannulated for intravenous injections of the anesthetic agent. The left common carotid artery was cannulated for AP and HR measurement. A circular arterial balloon-occluder (Fine Science Tools, Vascular Occluder-VO-1.5N/3.5 mm x1.5 mm) was secured around the descending thoracic aorta which was used to manually elevate AP as needed. After practice, the level of MAP increase and the rate of increase could be controlled at a constant level. Animals were placed in a stereotaxic instrument equipped with a head holder adapted to permit the neck to be sharply flexed. A dorsal incision was made over the neck muscles, which were retracted to expose the atlantooccipital membrane. This membrane was opened with an incision, exposing the cisterna magna and the dorsal medulla. The rostral end of the area postrema was used as a rostrocaudal reference for stereotaxic coordinates [17–19].

### AP and heart rate

**Fig 1:**
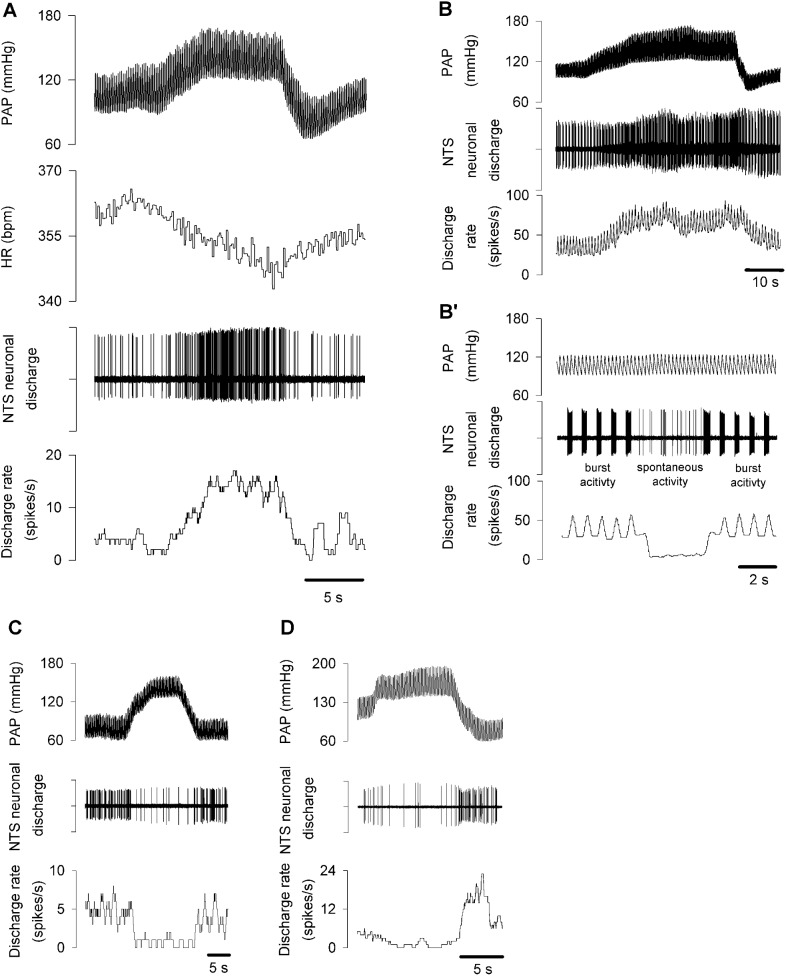
NTS putative barosensitive NA in response to AP changes. **A)** A representative excitatory neuron increased discharge rate in response to AP elevation. HR decreases in response to AP elevation. **B)** A pulmonary-related neuron. A representative excitatory neuron increased discharge rate during AP elevation. **B’)** The same neuron as in panel **B**. The basal burst discharge of this neuron had a firing pattern which was synchronized with the rhythmic ventilation. When the ventilator was turned off for 3 s, this neuron lost the burst activity and instead spontaneous activity appeared. The burst activity reappeared as the ventilator was turned on again. **C)** A representative NTS neuron decreased discharge rate during AP elevation. **D)** A representative NTS neuron had a bi-phasic response: first a decrease in discharge rate during AP elevation, then an increase in discharge rate during the descending phase of AP. PAP: pulse arterial pressure; HR: heart rate. S1 Fig.

The blood pressure catheter in the left common carotid artery was connected to a blood pressure transducer (iWorx/CB Sciences, BP-100). The transducer was positioned at the heart level. AP was measured using a Powerlab Data Acquisition System (AD Instruments, PowerLab 8/30, Chart 5.3 software) and displayed on the first channel (**Fig 1A,** PAP). The HR was calculated from pulse pressure in the first channel using the Ratemeter function and displayed on the second channel (**Fig 1A,** HR). Basal MAP and HR were recorded by averaging AP values and pulses for 2 min before elevation of AP.

### Extracellular recording of the NTS neurons

Extracellular recording of the NTS neurons was similar to what has been described in previous studies [17–19, 21, 22]. Briefly, a single-unit extracellular recording was obtained using a beveled glass micropipette (resistance 5–12 MΩ) filled with KCl (1M) as used before [21, 22]. With respect to calamus scriptorius, the right dorsal medial NTS was located 300 μm–600 μm rostral, 300 μm–500 μm lateral, and 300 μm–600 μm ventral. Using a Microdrive, the electrode was advanced into the dorsal medial NTS at a speed of 2 μm/s until spontaneous action potentials from a single unit were recorded. When action potentials were recorded, the electrode was maintained at the position for a couple of minutes, then the electrode was withdrawn at a speed of 2 μm/s for several μm to reduce possible contacts of the electrode with the neurons during AP elevation. The action potentials (APs) were amplified using a high impedance preamplifier (band pass 100–3,000 Hz) and fed into a window discriminator that generated a standard pulse for each spike. Potentials were visualized on an oscilloscope. The pulse output of the discriminator was then fed into a rate/interval monitor (HFC) whose analog output is proportional to the number of spikes per unit time. These signals were displayed online on the computer and recorded on the data acquisition system. The sampling rate was 10,000 measurements per second [17–19].

Baroreceptors were activated by the partial occlusion of the descending thoracic aorta to evoke responses from NTS barosensitive neurons [17–19]. The putative NTS barosensitive neurons were identified by their increased or decreased activity (frequency of action potential or discharge rate) relative to the basal activity in response to transient AP elevation by occlusion for 12 s. For each neuron tested, we repeated AP elevation procedure and measured the response again to ensure that the responses could be replicated. We did not analyze the data if we recorded a second unit. In addition, we did not analyze the neurons in which the amplitude of the action potential became abnormally larger, but a minimal variation of amplitude was acceptable [11]. Some neurons that were inhibited by aortic occlusion were also encountered in the present study. Since our goal is mainly to study the barosensitive neurons with excitatory response to AP elevation, we did not further analyze the neurons with inhibitory or biphasic responses. As a note, we will discuss the technical concerns regarding whether the recorded NTS neurons in response to descending aortic partial occlusion-induced AP changes are barosensitive neurons in Discussion section.

### Sigmoid logistic function curve

During aortic occlusion, MAP, HR and NTS neuronal activity (NA) changes relative to the basal activity (% baseline) were measured. The NTS barosensitive neurons’ activity (NA)-ΔMAP relationship curve was fitted by logistic function [15–16, 23]. NA in response to MAP elevation was plotted as the percent change relative to basal discharge using SigmaPlot 11: **Y** = **A**/{1 + exp[-**B**(**X—X**_**50**_)]} + **Y**_**min**_, where **Y**_**min**_ is the minimum value of % NTS NA relative to the basal activity, **A** is the range of % NTS NA (Maximum- Minimum), **X**_**50**_ is the pressure at the midpoint of the range, and **B** is the slope coefficient. The peak slope [or Max gain (**Gain**_**max**_)] was determined by: **Gain**_**max**_ = (A)(B)/4 (%/mmHg) and was used to evaluate the sensitivity of the NTS neuronal response to changes in MAP. Pressure threshold **P**_**th**_ was calculated as X_50_ - (1.317/B), and pressure saturation **P**_**sat**_ was calculated as X_50_ + (1.317/B), respectively. For late neurons, HR reduction-NA relationship was fitting by the logistic function: **Y** = **A**/{1 + exp[-**B**(X—X_50_)]} + **Y**_**max**_, where **Y**_**max**_ is the maximum reduction of the HR (bpm), **A** is the range of HR response (Minimum-Maximum), **X**_**50**_ is the % NA at the midpoint of the curve, and **B** is the slope coefficient. Max gain **Gain**_**max**_ = (A)(B)/4 and was used to evaluate the sensitivity of the HR reduction to NA changes. HR reduction threshold **P**_**th**_ was calculated as X_50_ + (1.317/B) and HR reduction saturation **P**_**sat**_ was calculated as X_50_ - (1.317/B), respectively.

### Statistical analysis

The average of MAP, HR, firing rate and the parameters in the logistic function were calculated. The data were presented as mean ± SE. Comparisons between groups were made using Student’s t-test or Two-way ANOVA repeated measures, Newman-Keuls post hoc analysis. p < 0.05 was considered significant.

## Results

### Putative NTS barosensitive neurons

Data were collected from 18 animals. The baseline level of MAP and HR were measured before occlusion. They were 99.6 ± 2.2 mmHg and 397.2 ± 6.4 bpm, respectively. Forty six NTS putative barosensitive neurons were analyzed. Thirty five of them increased firing upon MAP increase, and were thus considered excitatory (**Fig 1A and 1B**). Four excitatory neurons were pulmonary-related barosensitive neurons. These neurons had regular basal burst activity before the AP increase, which was synchronized with the artificial ventilation **(Fig 1B).** The regular basal burst activity was seen more clearly in **Fig 1B’.** When we suspended ventilation for 3 s, the neuron in **Fig 1B’** stopped exhibiting a respiratory pattern, and irregular spontaneous firing appeared. After ventilation was resumed, the regular basal burst activity reappeared, which indicates the respiratory nature of the neuron. Seven other neurons decreased firing upon MAP increases and returned to basal firing rate when MAP was reduced to the baseline levels, and were thus described as inhibitory (**Fig 1C**). Four additional neurons were biphasic: they decreased firing upon MAP increases, but then their firing rate increased as MAP decreased (**Fig 1D**).

### ΔMAP, ΔHR and firing rate of the excitatory neurons

The 35 excitatory neurons that responded to AP elevation were characterized. The mean MAP increase in these neurons was 37.5 ± 0.9 mmHg at a rate of 8.4 ± 0.3 mmHg/s and mean baroreflex bradycardia was 22.3 ± 2.6 bpm. The baroreflex sensitivity was 0.60 ± 0.07 bpm/mmHg (ΔHR/ΔMAP). Mean baseline firing activity was 7.1 ± 0.8 spikes/s and NA had a mean peak increase of 295.6 ± 35.3% relative to baseline.

### Early and late activation of excitatory neurons

**Fig 2:**
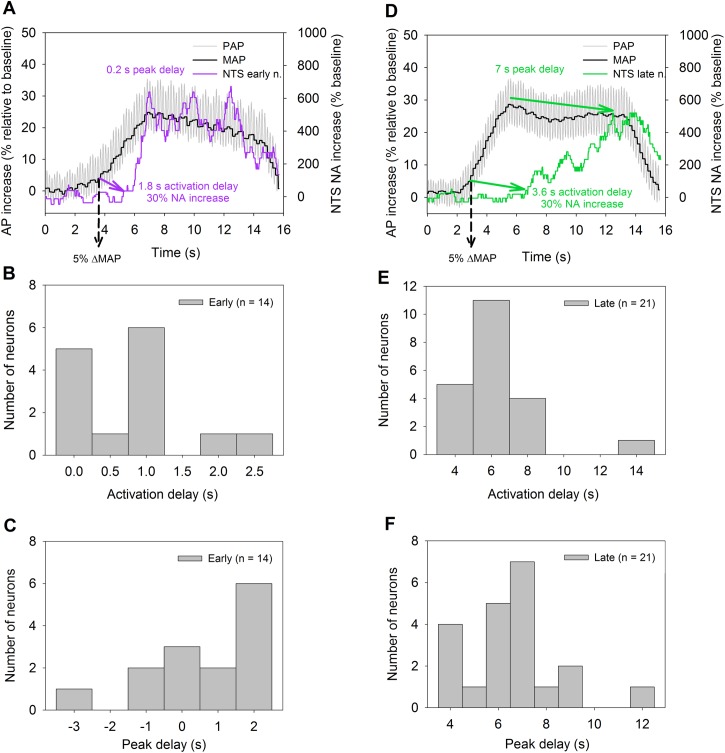
Activation and peak delays of NTS early and late NA. AP increase (%) relative to baseline and NA increase (%) relative to the basal activity during AP elevation are superimposed on each other. 5% of MAP increase (ΔMAP) was defined as the MAP threshold. 30% of NA increase was defined as the NA activation threshold. The time period from 5% of MAP increase to 30% of NA increase was defined as NA activation delay. The period from the ΔMAP peak to NA peak increase was defined as the peak delay. **A)** A representative early NA. The NA activation delay for this neuron was 1.8 s and the peak delay was 0.2 s. **B)** Distribution of the NA activation delay of early neurons. **C)** Distribution of the peak delay of early neurons. **D)** A representative NTS late NA. The NA activation delay for this neuron was 3.6 s and the peak delay was 7.0 s. **E)** Distribution of the NA activation delay of late neurons. **F)** Distribution of the peak delay of late neurons. The negative NA delay indicates the NA peak was before the ΔMAP peak. S2 Fig.

The majority of NTS excitatory neurons did not increase firing rates simultaneously with MAP increases. Fourteen neurons increased discharge rate during the early phase of the MAP ramp increase that ranged about 3–5 s (**Fig 2A**), However, 21 other neurons did not significantly increase discharge rate until the MAP reached near/after its peak plateau which lasted for about 5–7 seconds (**Fig 2D**). For the early neurons, the NA-ΔMAP relationship could be well fitted by logistic function curve (R^2^ > 0.90) (**Table 1**). The neurons that increased firing at the later stage and could not be fitted by logistic function curve were called the late neurons. All these neurons decreased firing rate during the release of the occlusion of the descending aorta.

**Table 1:** Parameters defining the early NA (% baseline)-ΔMAP logistic function curve

R^2^	A (range)	B (slope coficient)	X_50_	Y_min_	Gain_max_	P_th_	P_sat_
0.90 ± 0.01	352.5 ± 55.4	0.39 ± 0.05	32.4 ± 3.5	5.7 ± 5.8	29.3 ± 3.7	28.2 ± 3.7	36.6 ± 3.4

Values are means ± SE.

*n* = 14.

R: correlation coefficient; A: maximum − minimum (range); B: slope coefficient; X_50_: MAP at 50% of neuronal activity range; Y_min_: mininal neruonal activity; G_max_: maximum early neuronal activity gain (slope); P_th_: X_50_ − (1.317/ B); P_sat_: X_50_ + (1.317/ B). S2 File.

Excitatory neurons increased their firing rates in response to MAP increases at different times. To measure the time course of the responses, 5% MAP increase relative to basal MAP pressure was defined as the reference starting time point. A 30% increase of NTS neuron firing rate relative to basal firing rate was defined as the ΔMAP-induced threshold response of NTS neuron [17]. For 14 early neurons, the mean ΔMAP threshold was 14.12 ± 1.65 mmHg which evoked a 30% increase of NTS neuronal firing rate relative to basal rate. For 21 late neurons, the mean ΔMAP threshold was 34.07 ± 1.72 mmHg.

Early neurons were activated during MAP increase ramps. Early neuron activation delay was defined as from the time of 5% MAP increase to the time of 30% firing rate increase and ranged 0–2.5 s (**Fig 2B**). In contrast, the late neurons had the activation delay between 4–14 s (**Fig 2E**). The distribution of peak delays from the first MAP peak to the first NA peak firing rate for the early neuron (range: -3 to 2 s) is shown in **Fig 2C**, whereas the distribution of peak delay for the late neurons (range: 4 to 12 s) is shown in **Fig 2F**.

### The time-course of early and late NA

**Fig 3:**
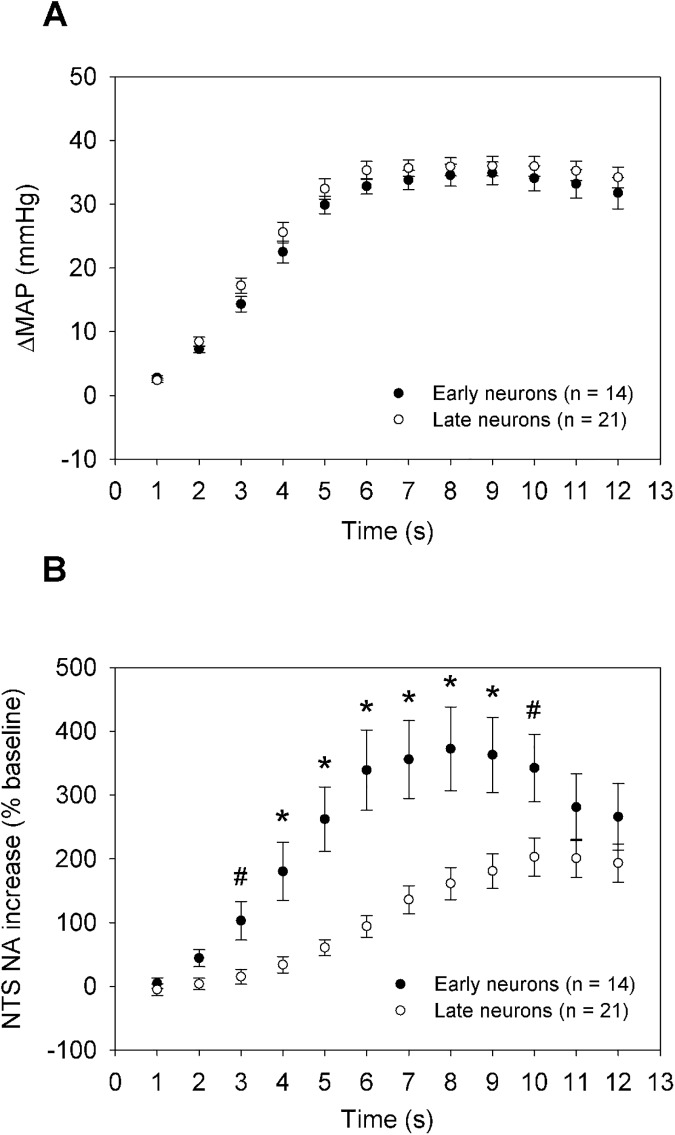
The time-course of NTS barosensitive NA increase (%, relative to baseline) in response to ΔMAP. **A)** During 12 s descending aorta occlusion, ΔMAP were similar for early and late neurons (p > 0.05). **B)** During 12 s descending aorta occlusion, NA increase of the early neurons had a significantly larger response in response to MAP increase than that of the late neurons (*p < 0.01, ^#^p < 0.05). S3 Fig.

To compare the differences of the NA in early and late neurons, we measured the time-course of NA responses to ΔMAP. NA was calculated as the percent (%) change relative to basal discharge. Change in MAP (ΔMAP) and NA were sampled every second for up to 12 seconds during occlusion-induced MAP elevation. The seconds 1 and 2 were defined at the first data points which were < 5% ΔMAP and > 5% ΔMAP, respectively. As shown in **Fig 3A**, ΔMAP was very similar in early and late neurons within 12 s. The rate of ΔMAP to the peak was also similar (early: 8.49 ± 0.6 mmHg/s, late: 8.48 ± 0.4 mmHg/s; p >0.05).

The basal neural activity (NA) of the early and late neurons was comparable (6.1 ± 1.3 vs 7.7 ± 1.1 spikes/s, p > 0.05). Since the rates of ΔMAP to the peak were also similar, we compared NA increases (%) and found that the early neurons had a significantly higher increase of NA than the late neurons at 3–10 s (p < 0.05). Noticeably, there was a trend for decline of NA within 8–12 s, but was not significantly different (p > 0.05) during 12 s AP elevation. The maximum NA increases (%) of early and late neurons were significantly different [400.2 ± 63.3% (early) and 225.8 ± 34.6% (late), p < 0.05].

### Early NA– ΔMAP sigmoid relationship

**Fig 4:**
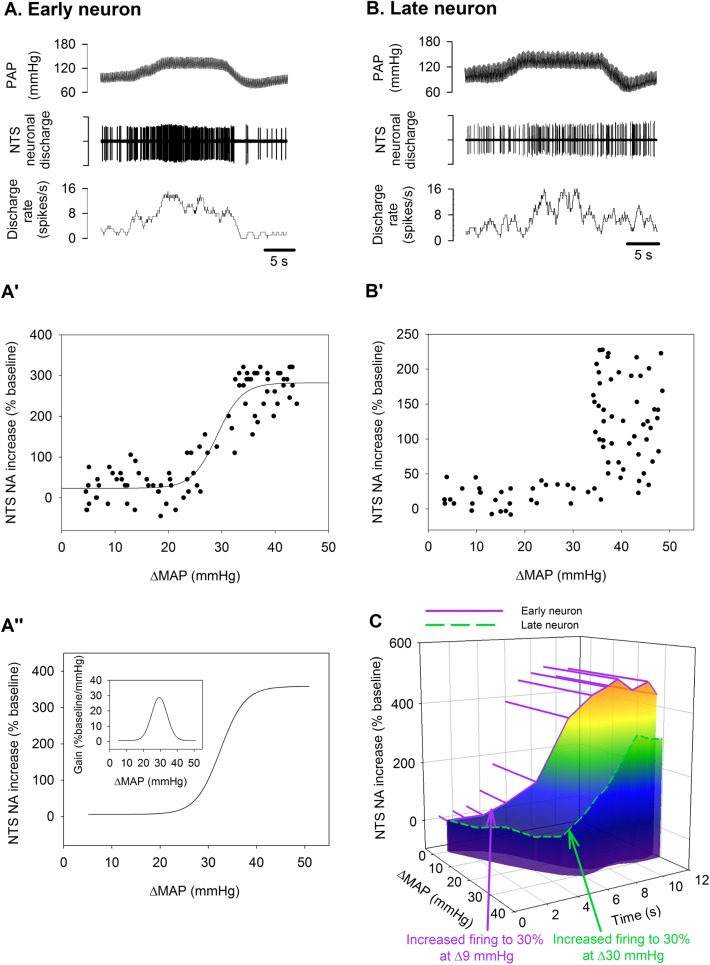
NTS neurons activity and ΔMAP relationship. **A)** Original recordings of a representative early NA during MAP increase. **A’)** The early NA- ΔMAP relationship curve was fitted using the sigmoid logistic function. **A”)** The composite early NA - ΔMAP relationship curve was reconstructed using the averaged parameters of the logistic function curves of the early neurons as shown in **Table 1**. **B)** Original recordings of a representative late NA during MAP increase. **B’)** This late neuron activity in reponse to MAP increase could not be fitted with the logistic function curve because it did not increase firing rate until ΔMAP reached the peak (about 35–40 mmHg). **C)** A 3-D plot shows the different charateristic firing patterns of these two representative early and late neruons. S1 File.

To characterize the NA-ΔMAP relationship, we used the logistic function curve. For early neurons, the NA increase-ΔMAP curve was well fitted by the logistic function curve (R^2^ > 0.90). **Fig 4A** shows the original recording of an early NA in response to MAP elevation. **Fig 4A’** shows a representative NA increase-ΔMAP curve of this neuron. The **Table 1** included the averaged parameters of the logistic function curve. **Fig 4A”** is the composite curve from the averaged parameters in **Table 1**. The inset of **Fig 4A”** shows the gain distribution curve that has a Gain_max_ of 29.3%/mmHg. For the late neurons, we could not construct a NA increase-ΔMAP curve because the NA did not change until the ΔMAP reached near/after the peak (**Fig 4B and 4B’**). A 3-D plot in **Fig 4C** shows the difference of the characteristic firing pattern of the two representative early and late neurons. The early neurons increased firing to 30% at 9 mmHg of ΔMAP at 2 s, but the late neurons increased firing to 30% at 30 mmHg of ΔMAP at 4 s.

### Early and late NA–HR reduction relationship

**Fig 5:**
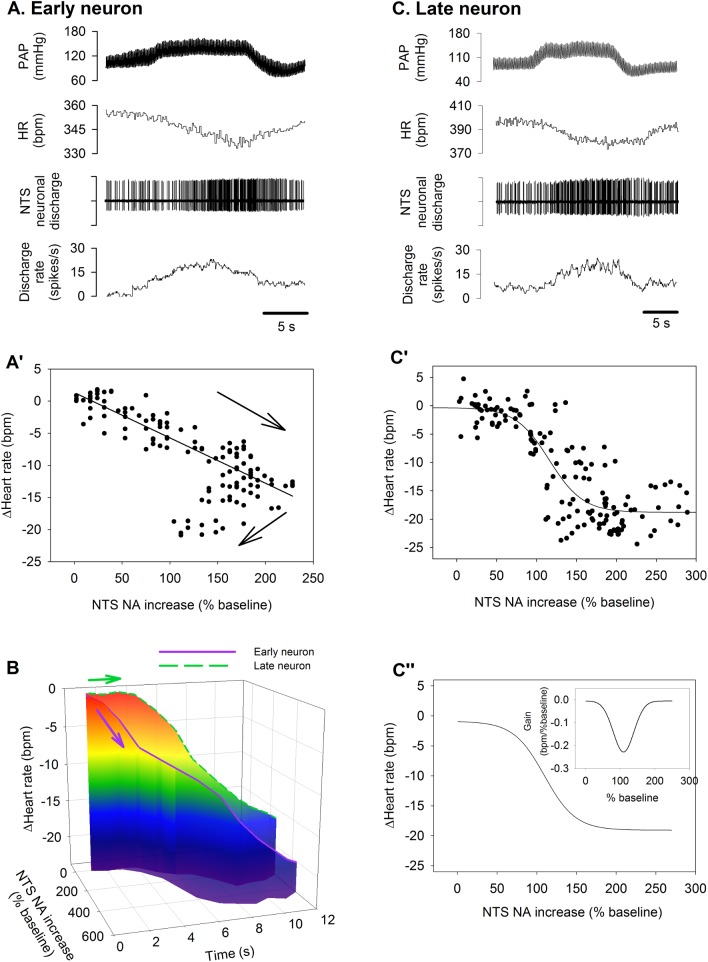
HR and NTS neurons activity relationship. **A)** Original recordings of a representative early NA and the HR reduction during MAP increase. **A’)** The initial HR reduction was linearly correlated with the increase of this early NA till the peak firing (about 200% relative to the baseline). After the peak firing, this neuron activity declined while the HR was still decreasing. **B)** A 3-D plot shows the difference of the HR reduction-NA between two representative early and late neurons as shown in **Fig 4C**. Whereas the early neuron contributed to the initial HR reduction, the late neuron did not contribute to HR reduction until a later time. **C)** Original recordings of a representative late neuron activity and the HR reduction during MAP increase. **C’)** The HR and this late NA relationship curve was fitted using the logistic function. **C”)** The composite HR-late NA relationship curve was reconstructed using the averaged parameters of the logistic sigmodal function curves of the late neurons as shown in **Table 2**.

For early neurons, the NA increase in the early phase of ΔMAP linearly correlated with the HR reduction (**Fig 5A and 5A’**). Since this early neuron showed some adaption during the sustained MAP elevation (**Fig 5A**), NA decreased during the late phase of ΔMAP, while the HR continued to decrease (**Fig 5A’**). To show the HR reduction correlation with NA more clearly, a 3-D plot in **Fig 5B** shows the HR reduction-NA relationships during a 12 s aortic occlusion from the two representative early and late neurons used in **Fig 4C**. The early neuron correlated to the HR reduction during 12 s occlusion, whereas HR reduction did not correlate with the late NA in the initial phase of ΔMAP but correlated well with the HR decrease in the late phase of ΔMAP (**Fig 5B**). **Fig 5C** was another late neuron which also shows that the HR decrease correlated with late NA. HR reduction-late NA relationship could be well fitted using the sigmoid logistic function curve. The curve of this representative late neuron is shown in **Fig 5C’**. The composite curve of the 21 late neurons is shown in **Fig 5C”** using the mean parameters in **Table 2**. The inset of **Fig 5C”** shows the Gain distribution curve with a Gain_max_ of -0.23 ± 0.04. Thus, both early and late neurons contribute to HR reduction, but in a different manner: the early neurons may contribute to the HR reduction at the initial and later phase of 12 sec occlusion, whereas the late neurons may contribute to the later phase HR reduction during sustained MAP elevation.

**Table 2:** Parameters defining heart rate reduction (ΔHR)-late NA (% baseline)-logistic function curve.

R^2^	A (range)	B (slope coefficient)	X_50_	Y_max_	Gain_max_	NA_th_	NA_sat_
0.75 ± 0.01	18.9 ± 1.2	-0.04 ± 0.006	111.8±18.4	-21.2 ± 1.3	-0.23 ± 0.04	67.3 ± 12.9	156.3 ± 24.3

Values are means ± SE.

*n* = 21.

R: correlation coefficient; A: minimum- maximum (range); B: slope coefficient; X_50_: NA at 50% of HR reduction range; Y_max_: maximum HR reduction; G_max_: maximum HR reduction gain (slope); NA_th_: X_50_ + (1.317/ B); NA_sat_: X_50_ - (1.317/ B). S2 File.

## Discussion

In this study different populations of putative NTS barosensitive neurons were identified. The majority of the neurons increased their firing rates in response to AP elevations, whereas other neurons decreased firing rates or had biphasic responses. The heterogeneous firing patterns are consistent with other reports [8–12]. The neurons with the increased firing rates were further classified into early and late neurons according to their increase of firing rates either during the MAP elevation ramp (early) or near/after MAP reached peak (late). Using the time-course comparison, we found that the early neurons had a significantly higher mean firing rate than the late neurons. For the early neurons, but not for the late neurons, the NA-ΔMAP relationship curve could be well fitted and quantitatively characterized by six parameters of the sigmoid logistic function curve. Finally, the relationship of early and late neurons activity with HR reduction was characterized. Apparently, the early neurons contributed to HR reduction overall in the period of AP elevation (< 12 s). In contrast, the late neurons activity did not contribute to the initial HR reduction but was correlated to the sustained MAP elevation-induced HR reduction. Therefore, both early and late neuron activity may contribute to HR reduction, but differently.

### Technical concerns

In the present study, we recorded NTS neurons in response to MAP elevations induced by partial occlusion of the descending aorta in anesthetized rats. There were a couple concerns. First, were these truly NTS baroreceptor-activated neurons? Partial occlusion of the descending aorta may increase the MAP in the upper portion of the body as measured through the left common carotid artery. Since arterial baroreceptors are localized predominately in carotid sinus and aortic arch [24], the recorded NTS neurons with excitatory response were most likely modulated by the arterial baroreceptors in carotid sinus and aortic arch. However, partial occlusion of the descending aorta might have also changed the blood volume (pressure) within the ventricles, atria and pulmonary vessels, thus activating cardiopulmonary stretch receptors (cardiopulmonary mechanoreceptors). Previously, Hines et al. indeed showed that NTS neurons could respond to bolus saline injection-induced intra-atrial pressure, but spontaneous activities of these neurons did not correlate with fluctuations in atrial pressure [25]. In contrast, Seagard et al. reported another population of NTS neurons which all exhibited pulse-synchronous activity and were driven by vagal afferent nerve rather than by aortic depressor nerve and carotid nerves [26]. Presumably, these were cardiac mechanoreceptors [26]. These neurons mildly increased their firing activities following an increase in MAP, possibly due to the increase of pressure within the heart. Though upper body AP increase may potentially activate cardiopulmonary mechanoreceptors, the chances might be lower than the activation of baroreceptors in the aortic arch and carotid sinus. Compared to the aortic arch, there are much less vagal cardiac afferent mechanoreceptors (flower-sprays endings, end-nets and free terminals) in the atria than those in the aortic arch [27–30]. In addition, we failed to find any vagal afferent flower-sprays endings and end-nets in ventricles (unpublished observations). Since the firing pattern of the neurons found in our present study were very similar to those found in Zhang and Mifflin who used electrical stimulation of the aortic depressor nerve to identify NTS barosensitive neurons and PE injection to activate baroreceptors [11] we believe that the NTS neurons that we had recorded in this study were most likely activated by the baroreceptors in the aortic arch and carotid sinus. Considering all these possibilities, we here used “***putative****”* NTS barosensitive neurons which may include all baroreceptors (stretch receptors in nature) in the aortic arch and carotid sinus as well as mechanoreceptors in the heart and lung which can be activated by blood volume or blood pressure changes.

The second question concerns whether the recorded putative NTS barosensitive neurons were monosynaptic or polysynaptic neurons. Previously, direct electrical stimulation of aortic depressor nerves (ADN) was used to first identify monosynaptic or polysynaptic neurons[11–13]. Then, the discharge patterns of NTS barosensitive neurons were characterized in response to vasoconstrictor phenylephrine (PE)- /vasodilator sodium nitroprusside (SNP) (i.v.)-induced AP increase/decrease in the whole body, or more specifically in response to pressure elevation-induced by a balloon-tipped catheter occlusion of the descending aorta. In the present study, we did not identify monosynaptic or polysynaptic neurons to classify neurons. Instead, we selected excitatory NTS neurons because they were the majority of the neurons and activation of these neurons correlated with baroreflex bradycardia. Among these excitatory NTS neurons, it was apparent that some neurons responded faster than the others. Thus, we further classify these excitatory neurons into early and late neurons. Actually, the firing patterns of the early and late neurons could be seen in several other reports as discussed below, although they did not use the term “early” and “late” neurons [10–12]. Monosynaptic and polysynaptic neurons may have the firing patterns of early and late neurons (see below discussion). In the present study, we grouped the neurons according to firing patterns (early and late) rather than the monosynaptic or polysynaptic classification. We assumed that the NTS neurons with the same firing pattern may have similar functions. In below, we also discussed the relative contributions of the early and late neurons to baroreflex bradycardia.

### Early and late putative barosensitive neurons

The majority of neurons (35 out of 46) increased firing upon AP elevation. In contrast, 7 neurons decreased firing. Additionally, 4 neurons appeared to have a bi-phasic response: decreased firing rate upon AP elevation but increased firing rate following AP decrease. Similar types of NTS barosensitive neurons were reported by Zhang and Mifflin who also found that the NTS neurons increased discharge rate, decreased discharge rate or were bi-phasic in response to MAP elevation [11], as well as other neurons which increased or decreased firing rate in response to a SNP-induced MAP depression. Seagard et alalso reported some neurons with decrease in firing rate in response to carotid MAP elevation[31].

We observed that 14 neurons (out of 35) increased firing rate during the early phase of AP increase (early neurons; **Fig 2A and Fig 4A**), whereas the remaining 21 neurons increased firing rate only near/after AP elevation reached its peak (late neurons; **Fig 2D and Fig 4B**). Using the sigmoid logistic function, we fitted the data (firing rate % vs ΔMAP) from all excitatory neurons. If the data could be well fitted by the sigmoid logistic function, then they were accepted as the early neurons. Compared to the late neurons, the early neurons had much shorter activation and peak delays.

In examination of the previous original recordings of the other studies, we found that the firing patterns of these early and late neurons matched the major firing patterns in both monosynaptic and polysynaptic neurons. For examples, Zhang and Mifflin found that the majority of monosynaptic and polysynaptic NTS neurons (38 out of 58 neurons recorded) were excitatory and may increase the firing rate to MAP increase induced by PE injection [11]. In 24 monosynaptic excitatory neurons (MSN), the peak increases in discharge frequency occurred either before/during (5 MSNs) or after (19 MSNs) [11]. These 5 MSNs were very likely the early neurons, whereas other 19 MSNs were likely late neurons (e.g., see Figs 2A and 3A (early), and Fig 2B and Fig 4 (late) for MSN responses in [11]): the firing patterns of the neurons in their Figs 2A and 3A are similar to the firing patterns of our early neurons, and the firing patterns of the neurons in their Fig 2B and Fig 4 are similar to the firing pattern of our late neurons). Whereas in 19 polysynaptic excitatory neurons (PSN), the peak excitatory responses occurred either before/during (5 PSNs) or after (14 PSNs), thus these 5 PSNs were very likely the early neurons and the other 14 PSNs were the late neurons(see Fig 5A (early) and Fig 5B (late) for PSN responses in [11]). In those original figures for MSN and PSN early neurons, the neurons did not increase their firing rates until the MAP had increased up to about 20–40 mmHg [11]. Previously, Rogers et al also identified presumed secondary NTS neurons using ADN electrical stimulation and used PE to increase AP [10]. They observed that some neurons responded during the rising phase of MAP, but a delay of increase in neuronal firing rates were often observed (e.g., see Figs 3 and 4 in [10]): the activities of these neurons did not increase until about a 20–30 mmHg increase in MAP was recorded. Such a delay of increase in neuronal firing rate was also clearly seen in Deuchars et al who physiologically identified and anatomically labeled arterial baroreceptive NTS neurons in the working heart-brainstem preparation [12]. In Deuchars et al [12], they characterized baroreceptive NTS neurons in response to stimulation of the aortic arch and/or ipsilateral carotid sinus by an aortic balloon or by injection of perfusate via a catheter placed in the common carotid artery. In 7 morphologically labeled NTS barosensitive neurons, some of them could be classified as early neurons and others late neurons according to our strategy. Even for early neurons, they may not increase firing rate until about 25–40 mmHg pressure increase (e.g., see Figs 3, 7, and 8 in [12]). For late neurons (see Figs 4 and 5 in [12]), they did not increase firing rates until the pressure reached about the peak about 50 mmHg. Therefore, there is a significant time delay between MAP increase and the neuronal firing increase. For 14 early neurons in our study, the mean ΔMAP threshold was 14.12 mmHg and activation delays ranged from 0 to 2.5 s.

Whether early and late neurons receive different baroreceptor inputs is another issue. In our study, we had less early neurons than late neurons. Previously, we injected tracer into the nodose ganglia in order to anterogradely label depressor nerve innervation of the aortic arch, and observed that there were fewer large aortic depressor nerve fibers than small fibers [27–28, 30]. This observation is consistent with Brown [32], who reported that there are less A-fibers than C-fibers in the depressor nerve. Since C-type axons have much higher thresholds than A-fibers [32], it is likely that the early neurons may receive A-fiber inputs and the late neurons may receive C-fiber inputs. Previously, Seagard et al. reported that there are two types of baroreceptors, which have different activation thresholds [33]. Later, two types of NTS barosensitive neurons were identified [31]: rapidly adapting neurons which have a sudden onset firing after the pressure reaches a threshold value and begin to adapt in firing as the pressure continues to increase up to 250 mmHg, whereas the slow-onset non-adapting neurons have a much higher threshold than the other type and continuously and slowly increase their firing rate as the pressure increases to 250 mmHg. Recently, Chen et al reported that there was actually a continuum of activation thresholds of aortic baroreceptors [34]. In our study, we found that there is a continuum of activation delays for early and late neurons (**Fig 2**). Therefore, the NTS neurons which increase firing at different pressure thresholds may echo the distribution of activation thresholds of primary baroreceptor depressor nerves [34].

Another possible mechanism for the early and late neurons may be the NTS neurons receiving suprathreshold and subthreshold baroreceptor afferent inputs. NTS (both monosynaptic and polysynaptic) barosensitive neurons may receive suprathreshold and subthreshold aortic nerve inputs [13]. While suprathreshold neurons can increase firing during aortic nerve stimulation, the NTS subthreshold neurons would only increase firing in response to aortic nerve inputs after application of excitatory amino acid (EAAs) to these neurons. EAAs from baroreceptor afferent inputs as well as other peripheral and central sites may contribute to the integration within NTS. It is likely that some NTS neurons (secondary and higher order neurons) may receive subthreshold baroreceptor inputs and they are in a “reserved state” and can be activated and become suprathreshold neurons under certain condition [13]. Thus, we postulate that the early neurons may be correlated with suprathreshold neurons, whereas the late neurons may be correlated with subthreshold neurons. This is particularly interesting because acute short-term small fluctuations of AP pressure can trigger the firing of NTS suprathreshold neurons to adjust HR and AP, but prolonged AP elevation (e.g., chronic hypertension) may activate the reserved subthreshold or late neurons to further reduce the HR and lower the AP [13, 14]

### Early NA-MAP elevation sigmoid relationship

The relationship between baroreceptor depressor nerve activity and MAP has been well established and used [15, 20, 23]. But to our knowledge, the NTS NA-ΔMAP relationship curve has not been shown. In this study, we used the sigmoid logistic function to construct the NA-ΔMAP relationship in early NTS neurons. The characteristics of NTS NA in response to ΔMAP can be depicted by 6 parameters in **Table 1**. Whether such a NTS NA-MAP elevation relationship can be obtained in other types of NTS neurons, such as the neurons whose firing rate decreases as MAP elevated or the neurons those firing rate increases as MAP is depressed, is an issue and should be further explored. For late neurons, we could not construct a NTS NA-ΔMAP relationship curve because the MAP had already reached the plateau before NTS neurons started to increase discharge from their basal activities. As shown previously [15, 16, 20, 35, 36], the sigmoid logistic curves can be used to study the drug- and disease-induced changes of baroreceptor depressor nerve function and sympathetic nerve functions. Using the sigmoid logistic function as a tool, we could differentiate early from late neurons. The early NA-ΔMAP logistic function curve can be a useful tool to compare the functional changes of early neurons in disease models.

### Time-course of the early and late NA-ΔMAP increase

To further characterize and compare the early and late neurons, we examined the time-course of their NA-ΔMAP increase. The time point was defined relative to the 5% increase of ΔMAP. Interestingly, the early neurons had higher firing rate increase in response to the MAP elevation compared to the late neurons during the period of AP elevation (< 12 s) before the occlusion was released. Time-course depicts the relationship between NA and MAP increase even after the MAP reached the plateau and neurons continued to increase discharge frequency. In contrast to the sigmoid logistic function which only applied to the early neurons, time-course measurement was applied to both early and late neurons. As shown in **Fig 3B**, the time-course can be used to compare NTS neurons activity between two types of neurons. Early neurons not only had a rapid activation, but also showed a higher firing rate in response to MAP increase than the late neurons. But we also noted that the activity of early neurons might decline from 8 to 12 s (Fig 3B), even though it was not significantly different within the time window <12 s.

The firing of the early neurons had a sudden-onset activity after the MAP reached the threshold which quickly reached the peak within 3–5 sec. This rapid increase of firing is somewhat similar to the sudden-onset neurons as reported before [8, 31]. But our neurons only show small adaptation. In contrast, the sudden-onset neurons showed much more significant adaptation to a large pressor ramp increase. This difference could be due to the difference in methodology: our MAP was first increased by 30–50 mmHg from the baseline MAP and then held relatively constant at 130–150 mmHg. In contrast, Seagard et al had a ramp carotid sinus pressure (CSP) increase from 0 to 250 mmHg and the neurons were silent up to a given CSP (67.8–98.1 mmHg) in dogs, and then they responded with burst of activity which immediately decreased as the ramp continued [8]. Our early neurons only showed the trend but did not show as much as adaptation possibly due to our relatively small increase of MAP compared to the large increase CSP [8, 31]

It is important to emphasize that the amount of MAP increase (ΔMAP) and the rate of ΔMAP to the peak was also similar in both groups during the 12 s aortic occlusion-induced MAP elevation since different MAP increases may lead to different responses from the same NTS neurons [10–11]. Also, we should mention that through practice, we could control the descending aorta occlusion such that the amount of MAP increase (ΔMAP) and the rate of ΔMAP to the peak was similar in both groups. One advantage is that it was more conveniently to increase pressure repeatedly, the basal AP and HR returned to the baselines much quicker after the release of aorta occlusion than PE injection.

### Early and late neuron activity-HR reduction relationship

Whether and how the firing of early and late neurons may contribute to the baroreflex bradycardia is an issue. In our study, we correlated early and late neurons activity with HR reduction. Based on the correlation, we suggested that the early neurons contribute to both the initial and late stages of HR reduction, whereas the late neurons contributed mainly to the late stage of HR drop during 12 s sustained MAP elevation. One concern might be that the above assumption is based on the correlation of early and late neuron activity with HR reduction. Although this correlation is not “causative”, it was a reasonable linkage, because it has been well established that activation of baroreceptor afferent results in activation of NTS barosensitive neurons and reduces HR. Application of excitatory amino acid glutamate, NMDA or non-NMDA receptor agonists into the NTS may increase the firing rate of NTS barosensitive neurons in a dose-dependent manner [37], microinjection of glutamate into the NTS in brainstem may reduce HR [38] and blockage of NMDA and non-NMDA receptors may attenuate baroreflex bradycardia [38–40]. Therefore, the correlation analysis of early and late neuron activities with HR was reasonable which may provide the valuable information that subgroups of NTS neurons with different thresholds may regulate the HR under different MAP conditions.

As shown in **Fig 3** and **Fig 5B**, it appears that the early neurons might be contribute to HR reduction more than the late neurons even during sustained MAP elevation, if we assumed that the high firing rate contributed more to HR reduction. However, this may not be true because if the MAP elevation had lasted even longer than 12 s, then the adaptation of early neurons could have become more significant and the firing rate of the early neurons would be less than the late neurons. In reality, it might be likely that more late neurons than early neurons are involved during high AP elevation. Thus, we should speculate that the early neurons may play a major role in the initial control of the HR and but reduce their contribution to long-lasting sustained high AP elevation. In contrast, the late neurons may not be activated to contribute to the initial control of HR, but are likely to be more responsive to long-lasting sustained high AP elevation. This assumption should be tested in the future.

## Supporting information

S1 Fig The data set to produce Fig 1. (JNB)

S2 Fig The data set to produce Fig 2. (JNB)

S3 Fig The data set to produce Fig 3. (JNB)

S1 File The data set to produce Figs 4 and 5 (JNB). (JNB)

S2 File Data Analysis for Tables 1&2 New 01.26.2017.xlsx (XLSX)
